# Diagnostic performance of image-guided vacuum-assisted breast biopsy after neoadjuvant therapy for breast cancer: prospective pilot study

**DOI:** 10.1093/bjs/znac391

**Published:** 2022-12-07

**Authors:** Elisabetta M C Rossi, Alessandra Invento, Filippo Pesapane, Eleonora Pagan, Vincenzo Bagnardi, Nicola Fusco, Konstantinos Venetis, Valeria Dominelli, Chiara Trentin, Enrico Cassano, Laura Gilardi, Manuelita Mazza, Matteo Lazzeroni, Francesca De Lorenzi, Pietro Caldarella, Alessandra De Scalzi, Antonia Girardi, Claudia Sangalli, Luca Alberti, Virgilio Sacchini, Viviana Galimberti, Paolo Veronesi

**Affiliations:** Breast Imaging Division, IEO European Institute of Oncology IRCCS, Milan, Italy; Breast Imaging Division, IEO European Institute of Oncology IRCCS, Milan, Italy; Breast Imaging Division, Radiology Department, IEO European Institute of Oncology IRCCS, Milan, Italy; Department of Statistics and Quantitative Methods, University of Milan-Bicocca, Milan, Italy; Department of Statistics and Quantitative Methods, University of Milan-Bicocca, Milan, Italy; Division of Pathology, IEO European Institute of Oncology IRCSS, Milan, Italy; Department of Oncology and Hemato-Oncology, University of Milan, Milan, Italy; Division of Pathology, IEO European Institute of Oncology IRCSS, Milan, Italy; Department of Oncology and Hemato-Oncology, University of Milan, Milan, Italy; Breast Imaging Division, Radiology Department, IEO European Institute of Oncology IRCCS, Milan, Italy; Breast Imaging Division, Radiology Department, IEO European Institute of Oncology IRCCS, Milan, Italy; Breast Imaging Division, Radiology Department, IEO European Institute of Oncology IRCCS, Milan, Italy; Division of Nuclear Medicine, IEO European Institute of Oncology IRCCS, Milan, Italy; Division of Medical Senology, IEO European Institute of Oncology IRCCS, Milan, Italy; Division of Cancer Prevention and Genetics, IEO European Institute of Oncology IRCCS, Milan, Italy; Department of Plastic and Reconstructive Surgery, IEO European Institute of Oncology IRCCS, Milan, Italy; Breast Imaging Division, IEO European Institute of Oncology IRCCS, Milan, Italy; Breast Imaging Division, IEO European Institute of Oncology IRCCS, Milan, Italy; Breast Imaging Division, IEO European Institute of Oncology IRCCS, Milan, Italy; Data Management, European Institute of Oncology IRCCS, Milan, Italy; Breast Imaging Division, IEO European Institute of Oncology IRCCS, Milan, Italy; Breast Imaging Division, IEO European Institute of Oncology IRCCS, Milan, Italy; Department of Oncology and Hemato-Oncology, University of Milan, Milan, Italy; Breast Service, Department of Surgery, Memorial Sloan Kettering Cancer Center, New York, New York, USA; Breast Imaging Division, IEO European Institute of Oncology IRCCS, Milan, Italy; Breast Imaging Division, IEO European Institute of Oncology IRCCS, Milan, Italy; Department of Oncology and Hemato-Oncology, University of Milan, Milan, Italy

## Abstract

**Background:**

Image-guided vacuum-assisted breast biopsy (VABB) of the tumour bed, performed after neoadjuvant therapy, is increasingly being used to assess residual cancer and to potentially identify to identify pathological complete response (pCR). In this study, the accuracy of preoperative VABB specimens was assessed and compared with surgical specimens in patients with triple-negative or human epidermal growth factor receptor 2 (HER2)-positive invasive ductal breast cancer after neoadjuvant therapy. As a secondary endpoint, the performance of contrast-enhanced MRI of the breast and PET–CT for response prediction was assessed.

**Methods:**

This single-institution prospective pilot study enrolled patients from April 2018 to April 2021 with a complete response on imaging (iCR) who subsequently underwent VABB before surgery. Those with a pCR at VABB were included in the primary analysis of the accuracy of VABB. The performance of imaging (MRI and PET–CT) was analysed for prediction of a pCR considering both patients with an iCR and those with residual disease at postneoadjuvant therapy imaging.

**Results:**

Twenty patients were included in the primary analysis. The median age was 44 (range 35–51) years. At surgery, 18 of 20 patients showed a complete response (accuracy 90 (95 per cent exact c.i. 68 to 99) per cent). Only two patients showed residual ductal intraepithelial neoplasia of grade 2 and 3 respectively. In the secondary analysis, accuracy was similar for MRI and PET–CT (77 *versus* 78 per cent; *P* = 0.76).

**Conclusion:**

VABB in patients with an iCR might be a promising method to select patients for de-escalation of surgical treatment in triple-negative or HER2-positive breast cancer. The present results support such an approach and should inform the design of future trials on de-escalation of surgery.

## Teaser

This single-institution prospective pilot study enrolled patients with breast cancer who underwent neoadjuvant therapy (NAT), had a complete response on imaging after NAT, and subsequently underwent image-guided vacuum-assisted breast biopsy before surgery. Some 18 of 20 patients showed a complete response (accuracy 90 (95 per cent exact c.i. 68 to 99) per cent). Only two patients had residual ductal intraepithelial neoplasia of grade 2 and 3 respectively. Accuracy was similar for MRI and PET–CT. These results from patients with triple-negative or human epidermal growth factor receptor 2-positive breast cancer should inform the design of future de-escalation trials in responders to NAT.

## Introduction

Neoadjuvant therapy (NAT) was introduced to increase the ability to perform surgery in patients with advanced or inflammatory breast cancer^1^. More recently, it has been used for selected patients with operable breast cancer, as it can downstage tumours, allowing breast-conserving surgery rather than mastectomy, with equivalent disease-free survival^2,3^. The pattern of response to NAT informs the tailoring of systemic and locoregional treatment, leading to escalation of treatment in non-responders and de-escalation in responders^4^. The ideal outcome of NAT is a pCR, which is associated with a favourable prognosis^5^. The rates of response to NAT have been shown to vary depending on disease subtype^6,7^. Among patients with triple-negative or human epidermal growth factor receptor 2 (HER2)-positive breast cancer, the rate of pCR after neoadjuvant therapy is 60–70 per cent^8–10^. It has been speculated that, for selected patients with a pCR after NAT, surgery can be omitted because the local tumour has already been eradicated.

Although breast-conserving surgery is associated with relatively low morbidity, it can negatively affect quality of life^11–13^. De-escalation of locoregional therapy after NAT would permit more use of conservation therapy as well as the possible omission of surgery^10,14–16^. To accurately identify candidates for de-escalation, better predictors of pCR are, however, needed^17^. In this setting, contrast-enhanced breast MRI and PET–CT have been shown to have good sensitivity and specificity for the prediction of a pCR^18–21^. Moreover, image-guided biopsy of the residual suspicious abnormality or the clip-marked tumour bed by vacuum-assisted breast biopsy (VABB) has performed well in the identification of a pCR^22–27^, with a low false-negative rate for the evaluation of pCR^27–29^. Ultrasound-guided VABB is easier to perform than MRI-guided VABB, less expensive, usually preferred by patients, and does not require the use of contrast media^30,31^.

The aim of this pilot study was to evaluate the concordance of pathology results between samples obtained by ultrasound-guided VABB and by surgery for the assessment of pCR in patients with an imaging complete response (iCR) after NAT.

## Methods

### Participants and study design

This single-institution prospective pilot study (NCT04365803) was conducted from April 2018 to April 2021 at the European Institute of Oncology, and was approved by the European Institute of Oncology Institutional Review Board and Ethics Committee on 28 February 2018 (R717/18-IEO 758). Eligible participants were women aged over 18 years with a diagnosis of triple-negative and/or HER2-positive invasive ductal breast cancer, of any tumour size (T1–T4) and lymph node involvement (N0–N3), without metastases (M0), who were candidates for NAT. Exclusion criteria were: multifocal, multicentric, and bilateral breast cancers; microcalcifications on mammography; diagnosis of associated ductal carcinoma *in situ*; and/or presence of chronic or psychiatric disorders.

For each participant, imaging assessment (including mammography, ultrasound, MRI, and PET–CT) was performed before and after NAT. An iCR was defined by the absence of any abnormality on imaging. A marker clip (UltraClip Breast Tissue Marker™; Bard Biopsy Systems, Tempe, AZ, US), if not present, was placed via an ultrasound-guided procedure before NAT in all participants. Although lymph node assessment was not an inclusion criterion, ultrasound-guided fine-needle aspiration was performed if suspicious lymph nodes were observed on imaging. Patients with an iCR after NAT underwent ultrasound-guided VABB to sample at the site of the marker clip (after confirming that there had been no clip migration), which represented the tumour bed.

Before inclusion in the study, all patients underwent an informed consent discussion and received a detailed explanation of the study aims, with the acknowledgment that the VABB procedure would not provide benefit. All included patients gave written informed consent to participate. All patients with a negative *BRCA* gene test underwent breast conservation surgery, providing preservation of free margins and optimization of cosmetic outcomes. A radio-guided occult lesion localization technique, which included the injection of a macroaggregate of ^99m^Tc–labelled human serum albumin, was used to localize the clip. Once resected, the specimen was analysed by X-ray to confirm the presence of the clip.

Finally, the histopathology report of the surgical specimen served as the reference standard. For the purposes of this analysis, a pCR was defined by the lack of any residual disease in the breast. A breast pCR in the tumour bed was characterized by the presence of oedematous stroma, with inflammatory cell and macrophage infiltration, and stromal fibrosis.

### Treatment and imaging assessment

Patients received NAT in accordance with Italian national guidelines (anthracycline- and/or taxane-based therapy with the addition of HER2-targeted therapy for patients with HER2-positive disease)^32^. Mammography was performed with bilateral craniocaudal, mediolateral, and mediolateral–oblique views in tomosynthesis (Senographe Essential, GE Healthcare, Chalfont St Giles, UK; 3Dimensions, Hologic Turin, Italy). Breast MRI was undertaken with the patient in the prone position by use of a 1.5-T scanner (Optima MR450w; GE Healthcare, Milwaukee, WI, USA) and a dedicated eight-channel breast coil. The protocol included an axial T2-weighted fast-spin echo sequence, an axial diffusion-weighted imaging sequence, and a dynamic study with three-dimensional T1-weighted gradient echo sequences acquired once before and four times after intravenous administration of 0.1 mmol/kg gadolinium chelate at 90 s of temporal resolution.

[^18^F]fluorodeoxyglucose (FDG) PET–CT (standard procedure) was performed before and after NAT^33^. Patients were instructed to fast for at least 6 h before the scan, and blood glucose levels were measured before injection of ^18^F[FDG]. Patients received an intravenous injection of 2.5–3 MBq/kg [^18^F]FDG, up to a maximum of 370 MBq (10 mCi), followed by a 60-min uptake. All patients underwent PET–CT in a dedicated tomograph validated for proper quantification and quality of the images recorded. An attenuation-corrected whole-body scan (base of skull to midthighs), with 2–2.5 min per bed position, starting 60 min after tracer injection, was acquired. All patients underwent low-dose CT for attenuation correction and anatomical correlation of PET findings. All PET–CT images were analysed using a dedicated workstation (Advantage; GE Healthcare, Milwaukee, WI, USA). To define the presence of residual disease, any focal non-physiological [^18^F]FDG uptake above the surrounding background activity was considered consistent with the persistence of a malignant lesion.

A complete response on PET–CT was defined by the complete disappearance of the pathological radiotracer uptake observed on the baseline scan. In addition to a complete response on PET–CT, an iCR was defined by the absence of residual disease on ultrasound, mammography, or breast MRI, in accordance with the American College of Radiology Breast Imaging Reporting & Data System (BI-RADS) criteria^34^.

Participants with an iCR underwent ultrasound-guided VABB under local anaesthesia by use of an EnCor Enspire system (Bard, Becton Dickinson, Franklin Lakes, NJ, USA) with needles ranging from 10 to 7G. The cores were radiographed to confirm retrieval of the marker clip. After ultrasound-guided VABB, a new marker was placed to guide subsequent surgery.

### Histopathological evaluation of vacuum-assisted breast biopsy and surgical specimens

Histological and immunohistochemical evaluation of pre-NAT biopsy samples was carried out as part of routine clinical practice. Tissue samples obtained from VABB and surgical specimens were examined by the same local pathologist (non-blinded setting). Biomarkers were tested and reported in accordance with the breast biomarker reporting guidelines (version 1.4.1.0) published by the College of American Pathologists in June 2021^35^, and in accordance with the updated recommendations from the International Ki67 in Breast Cancer Working Group^36^. A pCR was defined by the absence of residual invasive and *in situ* tumour cells in the surgical specimen (ypT0).

### Statistical analysis

The primary objective of the study was to evaluate the concordance of histopathological results between samples obtained by ultrasound-guided preoperative VABB and the surgical specimen (considered the reference standard) for the assessment of pCR in patients with an iCR after NAT. On the assumptions that an accuracy of 75 per cent or less (the null hypothesis of the study) would indicate that the procedure is not worthy of further investigation, and that an accuracy exceeding 95 per cent would indicate that it is worthy of further study, an optimal Simon two-stage design^37^ was used to allow early termination of the study. In the first phase, 11 patients were recruited. If there was concordance between the VABB and surgical samples (in terms of pCR) for at least 10 patients, another 11 patients would be enrolled, giving a total of 22 patients. In the second phase, if concordance was documented for at least 20 patients, preoperative VABB would be considered adequate and worthy of further investigation. On the assumption that the true accuracy was below 5 per cent, there was an 80 per cent chance of early termination of the study. If the procedure was truly accurate, the two-stage design would ensure an 85 per cent probability of reaching this conclusion. The type I error rate was set at 5 per cent.

As two patients with evidence of residual disease at VABB were at first included in the trial and subsequently excluded after the end of enrolment, the actual sample size was 20 patients rather than the planned 22. Clinicopathological characteristics were reported using descriptive statistics with absolute and relative frequencies for categorical variables and median (i.q.r.) or range for continuous variables.

The accuracy of VABB was calculated as the percentage of patients with a pCR at surgery relative to the number of patients with a complete response at VABB. The accuracy, sensitivity, specificity, false-negative rate, false-positive rate, positive predictive value (PPV), and negative predictive value (NPV) of preoperative breast MRI and PET–CT for the prediction of a pCR compared with histological evaluation of the surgical specimen was also calculated. Both patients with an iCR after NAT and those with residual disease at post-NAT imaging were included in this analysis. Accuracy, sensitivity (calculated as the ratio between true-positive (patients with residual disease both at imaging and surgery) and the number of patients without a pCR in the surgical specimen), and specificity (calculated as the ratio between true-negative (patients with a pCR both at imaging and surgery) and the number of patients with a pCR in the surgical specimen) of breast MRI and PET–CT were compared using McNemar’s test. Exact binomial 95 per cent confidence intervals were also reported for all percentages.

Analyses were performed using SAS^®^ software version 9.4 (SAS Institute, Cary, NC, USA).

## Results

### Patient characteristics

**Fig. 1:**
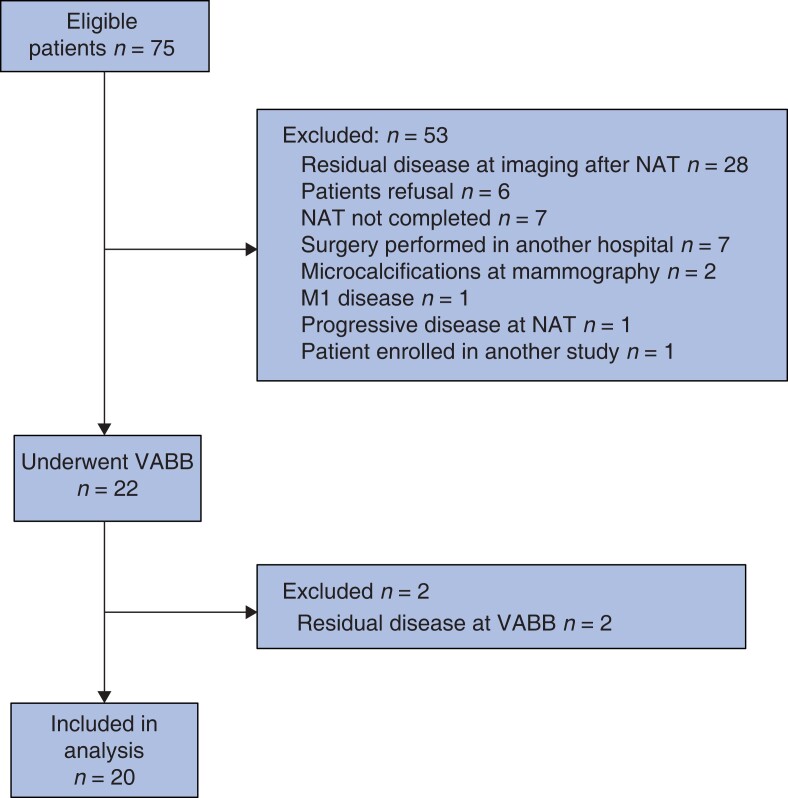
Study flow chart NAT, neoadjuvant therapy; VABB, vacuum-assisted breast biopsy.

Of the 75 eligible patients identified, 55 were excluded from the primary endpoint analysis. The reasons for exclusion are listed in the study flow chart (*Fig. 1*). In particular, two patients initially considered to be eligible were later excluded because revision of their mammography by radiologists at the authors’ unit revealed the presence of microcalcifications that were not reported in mammography performed elsewhere before enrolment. Residual disease was found at VABB for two patients. The remaining 20 patients, with a complete response after NAT both at imaging and VABB, were included in the primary analysis.

*Table 1* shows the characteristics of the 20 included patients (median age 44 (i.q.r. 35–51) years). Notably, 17 patients had triple-negative breast cancer, and three had HER2-positive breast cancer. *BRCA* mutation was found in four patients (three with *BRCA1* mutation and one with *BRCA2* mutation), whereas 11 patients did not have *BRCA* mutation. Genetic testing was not performed in five patients (25 per cent). Fine-needle aspiration of axillary lymph nodes was undertaken before NAT; five patients had a negative lymph node, whereas 15 had a positive lymph node. Intraoperative sentinel lymph node (SLN) biopsy was used as a standard of care. If positive, whether because of macrometastases, micrometastases or isolated tumour cells, axillary dissection was performed.

**Table 1:** Clinicopathological characteristics of study patients

	No. of patients (*n* = 20)
**Age (years), median (i.q.r.)**	44 (35–51)
***BRCA* mutation**	
ȃNo	11
ȃ*BRCA1*	3
ȃ*BRCA2*	1
ȃUnknown	5
**cT category**	
ȃcT1	2
ȃcT2	15
ȃcT3	3
**cN category**	
ȃcN0	5
ȃcN1	15
**Subtype**	
ȃTriple-negative	17
ȃHER2+	3
**Hormone therapy**	
ȃNo	16
ȃLHRH agonists	4
**Side**	
ȃRight	10
ȃLeft	10
**Surgery**	
ȃNipple-sparing mastectomy + SLN biopsy	7
ȃQuadrantectomy + SLN biopsy	13
No. of SLNs removed, median (range)	3 (1–11)

HER2, human epidermal growth factor receptor 2; LHRH, luteinizing hormone-releasing hormone; SLN, sentinel lymph node.

### Concordance between vacuum-assisted breast biopsy and surgical specimens

The median number of samples obtained by VABB was 10 (range 6–12). Patient tolerance of the VABB procedure was excellent, and there were no biopsy-related adverse events. At surgery, 18 of 20 patients showed complete a response (accuracy 90 (95 per cent exact c.i. 68 to 99) per cent). Only two patients had residual ductal intraepithelial neoplasia of grade 2 and 3 respectively.

### Imaging findings and accuracy of imaging for prediction of pCR

The maximum breast tumour size on pre-NAT imaging was less than 20 mm in two patients (9 per cent), 20–50 mm in 17 (77 per cent), and over 50 mm in 3 (14 per cent). Any focal non-physiological [^18^F]FDG uptake above the surrounding background activity was considered consistent with the presence of residual disease. Suspicious axillary lymph nodes were not observed on post-NAT imaging for any patient.

The imaging analysis included both the 22 patients with an iCR after NAT (two with residual disease at VABB, excluded from the primary analysis; 20 with a complete response at VABB, included in the primary analysis) and 28 patients with residual disease at post-NAT imaging who underwent surgery directly (*Fig. 1*). One of the two patients with residual disease at VABB, and six of the 28 with residual disease at post-NAT imaging were found to have a pCR at surgery.

*Table 2* shows the performance of breast MRI and PET–CT for prediction of a pCR in the surgical specimen. The overall accuracy was similar between breast MRI and PET–CT (*P* = 0.759). Breast MRI had a higher sensitivity (*P* = 0.031) but a lower specificity (*P* = 0.008) than PET–CT. The NPV was 79 (95 per cent c.i. 58 to 93) per cent for MRI and 71 (54 to 85) per cent for PET–CT. The PPV was 74 (52 to 90) and 100 (69 to 100) per cent respectively.

**Table 2:** Performance of MRI and PET–CT for the prediction of pCR at the time of surgery

	Breast MRI (*n* = 47*)	PET–CT (*n* = 45†)
Accuracy (%)	77 (62, 88)	78 (63, 89)
Sensitivity (%)	77 (55, 92)	50 (27, 73)
Specificity (%)	76 (55, 91)	100 (86, 100)
False-negative rate (1 – sensitivity) (%)	23 (8, 45)	50 (27, 73)
False-positive rate (1 – specificity) (%)	24 (9, 45)	0
Positive predictive value (%)	74 (52, 90)	100 (69, 100)
Negative predictive value (%)	79 (58, 93)	71 (54, 85)

Values in parentheses are 95% exact confidence intervals. The analysis included 50 patients: 22 with an imaging complete response (2 with residual disease at vacuum-assisted breast biopsy (VABB) and 20 with a complete response at VABB included in primary analysis) and 28 with residual disease at postneoadjuvant therapy imaging. *Three patients had missing information on MRI. †Five patients had missing information on PET.

### Surgery

After VABB, 13 patients (65 per cent) underwent radio-guided lumpectomy and SLN biopsy, and seven (35 per cent) had nipple-sparing mastectomy with SLN biopsy because of *BRCA* mutation (*n* = 3) or patient preference (*n* = 4). Intraoperative SLN showed no lymph node metastasis. No complications, such as haematoma or infection, were noted after the surgical procedure.

## Discussion

In the present study, NAT was chosen by the multidisciplinary team to downstage the cancer depending on the biological subtype, with the aim of providing less invasive surgery, reducing postoperative complications, and improving cosmetic outcomes. The expanded use and improved efficacy of NAT for the management of triple-negative and HER2-positive breast cancer have led to increasing numbers of patients with no detectable residual disease at the time of surgery^38^. In such patients, a pCR is associated with a low risk of locoregional recurrence and better overall survival^8,9,22,38,39^. It was shown here that ultrasound-guided VABB, used in concert with other imaging modalities, can reliably identify a pCR in patients with triple-negative or HER2-positive breast cancer.

In recent decades, the widespread adoption of NAT has facilitated increased use of breast-conserving surgery, instead of mastectomy, with equivalent survival outcomes. Although breast-conserving surgery is a procedure with relatively low morbidity, it still affects quality of life. Approximately 30 per cent of patients who undergo breast-conserving surgery and SLN biopsy report moderate, persistent pain 2 years after surgery, and patients who have breast-conserving surgery and mastectomy report similarly lower quality of life up to 8 years after surgery^38^.

In 2017, the St Gallen International Breast Cancer Conference highlighted opportunities for de-escalation of breast cancer treatment on the basis of tumour stage and tumour biology^40^. The omission of surgery should be restricted to patients for whom no residual disease after NAT can be expected. Therefore, patients who have a high probability of a pCR, and for whom there is evidence of concordance between iCR and pCR, are appropriate candidates for trials that omit surgery. Because NAT is highly effective in patients with triple-negative and HER2-positive breast cancer, with pCR rates exceeding 60 per cent^8–10,22,38,41^, the role of surgery may be limited to histopathological confirmation of a pCR. Surgery could be perceived to contradict efforts towards tailored treatments in these patients, as it exposes them to potentially unnecessary procedures with associated morbidity and psychological burden^41^. Currently, even with a pCR, radiotherapy is usually given after NAT and breast-conserving surgery^42^. A clinical trial that will follow the present pilot study will focus on an alternative local treatment with radiotherapy only in patients with a pCR after NAT and thus the omission of surgery.

As the currently available image-guided biopsy methods alone are not sufficiently accurate for assessment of pCR after NAT for patients with breast cancer^43^, combining them with other modalities, such as radiological and nuclear medicine imaging, for the assessment of pCR might help to reduce the false-negative rate to an acceptable level. In the present study population, multimodal imaging was used to evaluate response after NAT. The unique part of this study was that iCR was assessed not only by breast ultrasound imaging and mammography but also by breast MRI and PET–CT. In a recent meta-analysis^18^ of breast MRI and PET–CT for prediction of a pCR after NAT, breast MRI had a higher sensitivity (0.88, 95 per cent c.i. 0.78 to 0.94) than PET–CT (0.77, 0.58 to 0.90), and a slightly lower specificity (0.69, 0.51 to 0.83) than PET–CT (0.78, 0.63 to 0.88). Here, the overall accuracy for prediction of a pCR was similar between breast MRI and PET–CT (77 *versus* 78 per cent; *P* = 0.762), but MRI had a higher sensitivity (77 *versus* 50 per cent; *P* = 0.029), although with a lower specificity (76 *versus* 100 per cent; *P* = 0.011). The combination of breast MRI and PET–CT^18,21^ has been shown to be superior to the combination of ultrasonography, mammography, and clinical examination^44^. In the present study, combined breast MRI and PET–CT had good sensitivity and specificity for the prediction of a pCR.

Of importance, there is no consensus on the threshold of NPV that is adequate to prompt reduction of the extent of surgery, and histological examination of the surgical specimen is the only currently validated biomarker of survival^45–47^. Accordingly, the NPV of imaging in the present study (77 per cent for PET–CT and 88 per cent for breast MRI) is insufficient to recommend changes to therapeutic management. Nevertheless, in this study population, there were no false-negative results among patients who underwent VABB, which suggests that such an approach has the potential to accurately identify a pCR. A PPV of 74 (95 per cent c.i. 52 to 90) per cent was observed for breast MRI and 100 (69 to 100) per cent for PET–CT.

Image-guided biopsy of a residual abnormality or the tumour bed/marker clip after NAT has good performance for the assessment of pCR in selected patients with breast cancer^22–27^. In a meta-analysis of nine trials (1030 patients)^43^, the pooled sensitivity and specificity of imaging-guided biopsy after NAT for the assessment of pCR was 0.72 (95 per cent c.i. 0.61 to 0.81) and 0.99 (0.89 to 1.00) respectively. Subgroup analyses and meta-regressions showed that image-guided biopsy had a significantly higher accuracy in trials that considered pCR than in those that considered cCR. In a trial^27^ from MD Anderson, combined fine-needle aspiration and VABB had good performance in identifying residual disease after NAT (false-negative rate 5 per cent), and these results have been confirmed in larger multicentre trials^28,29^. Here, VABB had an accuracy of 95 (95 per cent c.i. 77 to 100) per cent with two false-negative results (false-negative rate 10 per cent), suggesting that this technique may reliably detect a pCR and identify patients who do not require further local treatment, such as surgery.

Ultrasound-guided VABB was performed here as a percutaneous biopsy. Ultrasound-guided VABB is accurate, safe, and allows faster acquisition of large tissue volumes than core needle biopsy. VABB permits retrieval of contiguous tissue specimens by use of a single insertion with a larger-gauge probe than core needle biopsy, resulting in reliability of the histological diagnosis nearly equivalent to that of open biopsy^48^. In the present study, the presence of microcalcifications was an exclusion criterion. Therefore, ultrasound imaging was used to guide the procedure, as it is known to be relatively simple and cost-effective, and, in expert hands, is highly effective for the visualization of breast lesions^30^. Moreover, compared with MRI, ultrasosonography is more widely available, cheaper, and does not require the use of contrast media^31,48^.

The use of VABB to confirm an iCR after NAT could allow surgery to be avoided in selected patients with breast cancer. Therefore, if VABB is proven to be safe and effective, it has the potential to decrease surgical complications, improve quality of life, and decrease healthcare costs^17,25,26^. Moreover, in patients with triple-negative or HER2-positive breast cancer who have a pCR after NAT, the risk of nodal disease ranges from 3 to 10 per cent^41,49,50^. Accordingly, with the risk of residual nodal disease sufficiently low, the omission of axillary surgery in selected patients with an iCR after NAT also merits investigation in future clinical trials.

Of note, the use of non-surgical therapy that relies on state-of-the-art image-guided biopsy for avoidance of surgery represents a somewhat radical shift in approach, so many questions need to be addressed. For instance, some have argued that elimination of surgery is associated with only a very small improvement in quality of life^38^ because, in patients with breast cancer who have an iCR after NAT, lumpectomy with SLN biopsy is a minimal procedure with a reoperation rate of 0.7 per cent, 30-day morbidity rate of 1.9 per cent, and complication rate comparable to that of VABB^38,51^.

In addition to the single-centre nature of the study, other limitations should be noted. The study population included only patients with triple-negative or HER2-positive breast cancer (as these are more likely to respond to NAT than luminal breast cancers)^52^, who account for only one- quarter of patients with breast cancer^53^. Other breast cancer types will be considered for inclusion in the continuation of this clinical trial. Moreover, image-guided biopsy to assess the presence of residual disease in the breast after NAT is not performed routinely and is not included in standard breast cancer management pathways. The procedure is operator-dependent and requires the expertise of a breast radiologist using standardized assessment protocols. Therefore, the results of this study could be implemented in referral centres only and, at this point, only in the context of clinical trials in appropriately defined and selected patients. Here, 6–12 samples per VABB, with an average of 10, were considered, which undoubtedly is a limitation as it lacks standardization and reproducibility. This was one of the reasons for the negative results of the NRG Oncology BR005 study^54^. Therefore, in future studies, the number of VABB samples will have to be standardized. Another critical issue involves histopathological VABB assessment of a non-tumour specimen, as it may have been taken from the former cancer or outside of this region (that is non-representative VABB owing to sampling error). Furthermore, axillary imaging cannot reliably identify a pCR, and lymph nodes after NAT were not pathologically evaluated systematically. Finally, it is unclear whether avoidance of a low-morbidity outpatient surgery is worth the anxiety and uncertainty caused by additional imaging and biopsies. Accordingly, recruitment may be a challenge in trials offering a non-surgical treatment arm. It is the authors’ opinion that future clinical trials investigating the omission of surgery in patients with a pCR by VABB warrant further discussion.

Imaging alone after NAT lacks sufficient sensitivity and specificity for prediction of a pCR. Breast MRI and PET–CT combined with VABB of the residual lesions showed high accuracy. Therefore, VABB may play a role in the identification of appropriate patients for omission of surgery and to safely spare women unnecessary treatment-associated morbidity. The results of this prospective pilot study support the use of VABB in patients with triple-negative or HER2-positive breast cancer after NAT, and should inform the design of future trials investigating de-escalation of surgery.

## Data Availability

The data presented in this study are available on request from the corresponding author.
